# A comprehensive review of Schisandrin B’s preclinical antitumor activity and mechanistic insights from network pharmacology

**DOI:** 10.3389/fphar.2025.1528533

**Published:** 2025-02-10

**Authors:** Yanhua Fang, Juan Pan, Piao Wang, Ruoyu Wang, Shanshan Liang

**Affiliations:** ^1^ The Key Laboratory of biomarker high throughput screening and target translation of breast and gastrointestinal tumor, Affiliated Zhongshan Hospital of Dalian University, Dalian, Liaoning, China; ^2^ Liaoning Key Laboratory of Molecular Recognition and Imaging, School of Bioengineering, Dalian University of Technology, Dalian, China; ^3^ Department of Oncology, Central Hospital of Liwan, Guangzhou, China

**Keywords:** *Schisandra chinensis*, Schisandrin B, antitumor, molecular mechanisms, network pharmacology

## Abstract

As an active constituent in the extract of dried fruits of *Schisandra chinensis*, Schisandrin B exhibits diverse pharmacological effects, including liver protection, anti-inflammatory and anti-oxidant. Numerous studies have demonstrated that Schisandrin B exhibits significant antitumor activity against various malignant tumors in preclinical studies, which is achieved by inhibiting cell proliferation and metastasis and promoting apoptosis. As a potential antitumor agent, Schisandrin B holds broad application prospects. This review systematically elaborates on the antitumor effect of Schisandrin B and the related molecular mechanism, and preliminarily predicts its antitumor targets by network pharmacology, thereby pave the way for further research, development, and clinical application.

## 1 Introduction

In recent years, the incidence and mortality of malignant tumors on a global scale continue to ascend without interruption. In 2022, there were approximately 20 million newly diagnosed cancer cases worldwide. According to demographic projections, by 2050, the number of new cancer cases will attain 35 million (Bray et al., 2024). Consequently, it is of utmost importance to identify effective treatment plans and discover new therapeutic targets for malignant tumors. Natural drugs boast abundant sources, diverse structures, and extensive activities. On this basis, the development of new antitumor drugs and prodrugs with superior efficacy has become a hot field for numerous tumor scholars at present (Ma L. et al., 2021). Among them, small molecule drugs originating from the active ingredients of natural drugs, which are diverse in variety and possess the characteristics of multiple targets, multiple signaling pathways, and extensive pharmacological activities, have obtained remarkable achievements in both antitumor drug screening and mechanism research, thereby providing new thoughts for discovering new tumor treatment strategies (Basmadjian et al., 2014; Chopra and Dhingra, 2021).

*Schisandra chinensis* (called bei-wuweizi in Chinese, *S. chinensis*) is a plant of the *Magnoliaceae* family. Its dried fruits are traditional Chinese medicines (Yang K. et al., 2022). Schisandrin B (Sch B) is the most notable dibenzocyclooctene-type lignan in *S. chinensis* (Nasser et al., 2020). At present, most of the research on Sch B focuses on its effects in protecting the liver (Hong et al., 2017; Służały et al., 2024), treating cardiovascular and cerebrovascular diseases (Chen et al., 2013; Duan et al., 2021), anti-inflammation (Ran et al., 2018; Ma Z. et al., 2021), anti-oxidation (Kopustinskiene and Bernatoniene, 2021). In addition, certain preclinical studies have indicated that Sch B possesses antitumor potential in liver cancer (Song et al., 2023; Yang and Wu, 2023), colorectal cancer (Pu et al., 2021; Co et al., 2024), breast cancer (Fei et al., 2020; Li W. et al., 2024), gastric cancer (He et al., 2022) and other cancer types through promoting tumor cell apoptosis, inhibiting tumor cell proliferation, invasion and migration. In this review, we introduce the physicochemical properties and pharmacokinetics of Sch B, systematically summarize its antitumor effects and mechanisms in different tumors through preclinical studies, analyze and predict the main targets and signaling pathways of Sch B in antitumor through network pharmacology, with the objective of providing references for its further research, development and clinical application.

## 2 Methods

In August 2024, the author carried out an extensive search in the PubMed database for relevant literature published from January 2001 to August 2024. By using a collection of keywords such as “*Schisandra chinensis*”, “Schisandrin B″, “Sch B″, “antitumor”, “cancer”, “molecular mechanisms” and “network pharmacology”, 806 articles were retrieved in total. When it came to the literature screening stage, strict inclusion criteria were carefully established. The literature to be incorporated was required to meet multiple conditions. In the section of “Sch B Physicochemical and Pharmacokinetic Properties”, the literature should show a high relevance to Schisandrin B and have a high citation rate. For other literature, a strong link to the antitumor effect of Schisandrin B was necessary. Among comparable literature, those with a relatively high impact factor and a high standing in the journal ranking were preferred. Additionally, recently published works were given priority. After this rigorous selection procedure, 74 articles were finally cited in this paper.

## 3 Schisandrin B: physicochemical and pharmacokinetic properties

Sch B (C_23_H_28_O_6_) manifests as white flaky crystals. It has a molecular weight of 400.46 and its melting point ranges from 120°C to 121°C. Sch B is insoluble in water but soluble in organic solvents such as ethanol and dimethyl sulfoxide (DMSO). Studies have indicated that following oral administration in rats, Sch B may possess enterohepatic circulation, predominantly accumulate in the liver, and it is mainly excreted through the kidneys in the form of metabolites. The pharmacokinetics of Sch B is related to gender, with the absolute oral bioavailability of female mice being significantly higher than that of male mice. Sch B, which is widely distributed in the ovary and adipose tissue, shows linear pharmacokinetic characteristics within a certain concentration range (10 mg/kg, 20 mg/kg and 40 mg/kg) (Zhu et al., 2013; Wang et al., 2018). Shao et al. demonstrated in a myocardial infarction mouse model study that the utilization of matrix metalloproteinase-sensitive peptide-modified PEGylated lipid nanoparticles for carrying Sch B can enhance its solubility and bioavailability (Shao et al., 2017). A study has confirmed that Sch B exerts protective effects on various normal cells including liver parenchymal cells, cardiomyocytes, neurons, renal cells, and umbilical vein endothelial cells. Different concentrations of Sch B (1.67 μg/mL, 5 μg/mL, 15 μg/mL) show no cytotoxicity to nerve cells M146L. Moreover, Sch B can significantly increase the viability of human proximal tubular epithelial cells (HK-2) at 2.5 μM, 5 μM and 10 μM (Zhu et al., 2012). In animal experiments, low, medium and high concentrations of Sch B (20 mg/kg, 40 mg/kg, 80 mg/kg) were respectively administered intragastrically to rat models of myocardial ischemia-reperfusion injury. The apoptosis of myocardial cells was inhibited and myocardial ischemia-reperfusion injury in rats was improved (Zhang et al., 2017). These findings are sufficient to indicate that Sch B is characterized by low toxicity and high safety.

## 4 Role of Schisandrin B in different tumors

In the investigations on the antitumor efficacy of Sch B, researchers have determined that Sch B predominantly exerts its tumor-suppressing function via mechanisms including inhibiting the proliferation, invasion, and migration of tumor cell lines, promoting the apoptosis of tumor cells, and suppressing tumor angiogenesis (Table 1).

**TABLE 1:** Antitumoral effects and molecular targets of Sch B in various cancers.

Tumor type	Cell line	Target	Effect	References
Liver cancer	Huh-7 SMMC-7721 HepG2 Hepa1-6	RhoA, ROCK1, hsp70↓ Caspase-3, ROS, Th1/Th2↑	Proliferation Migration Apoptosis Pyroptosis Autophagy	Wu et al. (2004), Tan et al. (2022), Song et al. (2023), Yang and Wu (2023)
Colorectal cancer	HCT116 HT29 SW620 Caco2	Bcl-2, SIRT1↓ CHOP, Bax/Bcl-2, Caspase-3, SMURF2, p-FAK↑	Apoptosis Proliferation Cell Cycle Migration	Li et al. (2019), Pu et al. (2021), Sana-Eldine et al. (2023), Co et al. (2024), Li et al. (2024b)
Breast cancer	MS MDA-MB-435S 4T1, MCF-10A MDA-MB-231 BT-549 MDA-MB-468	P-glycoprotein, VEGF MMP-9, VIM, NOX4, TGF-β1, NLRP3, IL-1β, p-STAT3↓ E-cad↑	Multidrug resistance Migration Invasion Apoptosis Proliferation	Liu et al. (2012), Zhang et al. (2013), Dai et al. (2018), Fei et al. (2020), Jing et al. (2020), Chang et al. (2024), Li et al. (2024b)
Gastric cancer	SGC-7901 BGC-823	p-STAT3, VEGF, E-Cad HIF-1a, PI3K, MMP-2, FAK↓	Proliferation Migration Invasion Autophagy Apoptosis	Li et al. (2021a), He et al. (2022)
Glioma	U251, U87 SHG-44	p-Akt, p-mTOR, MMP-9 HOTAIR, Bcl-2, CDK4↓ miR-125a-5p, Caspase-3↑	Migration Invasion Apoptosis	Jiang et al. (2015), Li et al. (2015), Qi et al. (2016), Jiang et al. (2017)
Gallbladder cancer	GBC-SD NOZ	Bcl-2, NF-κB Cyclin D1, CDK-4↓	Apoptosis Proliferation Cell Cycle	Xiang et al. (2014)
Non-small cell lung cancer	A549	VIM, VEGF, Cyclin D1, CDK-4↓ E-cad, p53↑	Migration Invasion Cell cycle	Lv et al. (2015), Cai et al. (2021)
Ovarian cancer	SK-OV-3	MMP-2, MMP-9↓	Angiogenesis VM Invasion Metastasis	Zhang et al. (2021)
Osteosarcoma	143B MG63 SaOS2 U2OS	Wnt/β-catenin, PI3K/Akt↓ miRgg708-5p↑	Proliferation Metastasis	Wang et al. (2020), Wang et al. (2022)
Prostate cancer	DU145 LNCaP	p-PI3K/AKT p-STA3/JAK2↓	Cell cycle Apoptosis	Nasser et al. (2019)
Melanoma	B16F10	Wnt/β-catenin↓	Cell cycle Migration Invasion	Chen et al. (2023)

### 4.1 Schisandrin B in liver cancer

Malignant liver tumors are among the most prevalent malignant tumors globally. The oncogenesis is typically accompanied by a dismal prognosis and a low overall survival rate. Among them, hepatocellular carcinoma (HCC) constitutes 80%–90% of primary liver cancer. It is the fourth leading cause of cancer-related deaths worldwide (Li X. et al., 2021). Multiple studies have demonstrated that Sch B can inhibit the progression of liver cancer cell lines in various ways. Inducing apoptosis of tumor cells is an important approach in tumor treatment. Researchers have found that the RhoA/ROCK1 pathway is involved in regulating the proliferation and migration of HCC cells (Lin et al., 2018; Zhang et al., 2020). Sch B promotes the apoptosis of Huh-7 cells and restrains the proliferation, migration and invasion capabilities of Huh-7 cells via the RhoA/ROCK1 pathway (Yang and Wu, 2023). Additionally, Sch B can downregulate heat shock protein 70 (hsp70) and upregulate the expression of Caspase-3 to induce apoptosis of human hepatoma cells (SMMC-7721) (Wu et al., 2004). Pyroptosis refers to inflammatory programmed cell death (Cookson and Brennan, 2001). The apoptosis of HepG2 cells induced by Sch B transforms to pyroptosis in the presence of NK cells, which also indicates that Sch B has the potential to be a combined partner for immunotherapy against HCC (Song et al., 2023). Autophagy is a regulatory mechanism that eliminates unnecessary or dysfunctional cellular components and recycles metabolic waste (Xia et al., 2021). At the early stage of tumors, autophagy is capable of inhibiting tumorigenesis. The research conducted by Siran Tan et al. indicated that Sch B led to an excessive accumulation of reactive oxygen species (ROS), thereby inducing autophagy and reducing the activity of Hepa1-6 (Tan et al., 2022).

### 4.2 Schisandrin B in colorectal cancer

Colorectal cancer, ranking as the fourth most prevalent cancer globally, was seldom diagnosed several decades ago. According to statistics, nearly 900,000 people succumb to colorectal cancer annually (Zhou et al., 2021). In the early stage of colorectal cancer, patients typically exhibit no symptoms. When patients present with symptoms such as anemia, stomach discomfort, or rectal bleeding, it often indicates that colorectal cancer has advanced to an advanced stage and transformed into an aggressive, malignant, and metastatic tumor. The advancements in the pathophysiology of colorectal cancer have provided a variety of treatment options for it, including targeted therapy (Kasprzak, 2023). Vanessa Anna Co et al. discovered that Sch B had induced apoptosis both *in vivo* and *in vitro* by interacting with C/EBP homologous protein (CHOP) and up-regulating its expression level. Additionally, it was verified that Sch B inhibits cell proliferation and tumor growth (Co et al., 2024). Sch B also indirectly regulates Smad ubiquitination regulatory factor 2 (SMURF2) by suppressing the protein expression of Sirtuin 1 (SIRT1) and restrains the growth and metastasis of colon cancer cells (Pu et al., 2021). Preclinical studies have indicated that colitis-associated colorectal cancer (CAC) is closely associated with ulcerative colitis (UC) (Zhang et al., 2023). Sch B can activate focal adhesion kinase (FAK) to enhance the protective effect of the intestinal epithelial barrier. Moreover, Sch B can affect the intestinal flora, which can effectively prevent UC and thereby achieve the purpose of preventing CAC (Li et al., 2019). In addition to having the potential to become a targeted therapeutic drug for colorectal cancer, Sch B is also promising in combination therapy. It is well known that the overexpression of epidermal growth factor (EGFR) and its related pathways are the key regulatory factors of colorectal cancer (Koustas et al., 2017). Panitumumab, a monoclonal antibody targeting EGFR, is utilized in the treatment of colorectal cancer. The combination of Sch B and panitumumab can induce apoptosis of colorectal cancer cell lines by activating caspase-3 and down-regulating B-cell lymphoma-2 (Bcl-2), and its cytotoxic effect is more remarkable than that of panitumumab when used alone (Sana-Eldine et al., 2023).

### 4.3 Schisandrin B in breast cancer

Breast cancer, a malignant disease originating in the cells of the breast, is currently one of the leading causes of cancer-related deaths among women worldwide (Akram et al., 2017). Chemotherapy is one of the most commonly employed methods for treating breast cancer. However, breast cancer stem cells exhibit multiple drug resistance to chemotherapy drugs. Weinan Li et al. fabricated pH-sensitive nanoparticles (Sch B/AP NPs) to reverse the multidrug resistance of breast cancer stem cells, presenting a novel approach for the treatment of breast cancer (Li W. et al., 2024). To address the problem of poor targeting efficacy of traditional chemotherapy drugs, Ming Jing et al. developed a PFV-modified epirubicin and Sch B liposome which showed a remarkable anti-breast cancer effect (Jing et al., 2020). There is also a type of F127-modified lipid-polymer hybrid nanoparticles carrying Sch B (Sch B-F-LPNs), which can significantly reduce the number of metastatic lung nodules and inhibit the lung metastasis of breast cancer to a certain extent (Fei et al., 2020). Furthermore, the mechanism through which Sch B inhibits the progression of breast cancer is of great concern. Research results indicated that Sch B significantly inhibited the lung and bone metastasis of 4T1 mouse breast cancer cells by inhibiting TGF-β-induced epithelial-mesenchymal transition (EMT), suggesting the targeted effect of Sch B on breast cancer metastasis (Liu et al., 2012; Zhang et al., 2013). Sch B hinders the progression of triple-negative breast cancer (TNBC) by suppressing the production of interleukin-1β (IL-1β) induced by nucleotide-binding oligomerization domain-like receptor family pyrin domain containing 3 (NLRP3) (Chang et al., 2024). Sch B can also restrain the phosphorylation and nuclear translocation of signal transducer and activator of transcription 3 (STAT3), induce cell cycle arrest and apoptosis, thereby inhibiting the growth, migration and clone formation of TNBC cell cells and exerting its anti-breast cancer effect through preclinical studies (Dai et al., 2018).

### 4.4 Schisandrin B in gastric cancer

Gastric cancer (GC) is one of the most prevalent malignant tumors in the digestive tract system. Due to the characteristics of easy metastasis, high recurrence rate, and chemotherapy resistance of advanced GC, the survival rate of patients with advanced GC is low (He et al., 2020). Therefore, it is of utmost importance to investigate the pathogenesis of GC and identify effective treatment regimens. Previous research has demonstrated the role of traditional Chinese medicine in the treatment of GC (Hung et al., 2017; Liu et al., 2017). Peng Yang et al. analyzed the functional gene network in the occurrence of gastric cancer through bioinformatics, screened target genes, and thus discovered that four potential active components of traditional Chinese medicine, including Sch B, were expected to become therapeutic drugs for GC (Yang P. et al., 2022). There are conclusive research findings suggesting that Sch B is capable of suppressing the proliferation, migration and invasion of gastric cancer cells both *in vitro* and *in vivo*, inducing autophagy of gastric cancer cells by restraining the phosphorylation of STAT3; and it can enhance the therapeutic efficacy of the chemotherapy drug 5-fluorouracil (5-FU) (He et al., 2022). Furthermore, certain researchers have successfully fabricated R8 (RRRRRRRR)-modified vinpocetine and Sch B liposomes. This targeted liposome is capable of inducing apoptosis in BGC-823 cells, inhibiting the metastasis of tumor cells, and strengthening the targeting effect on tumor cells, demonstrating remarkable antitumor efficacy both *in vitro* and *in vivo* (Li X.-Y. et al., 2021).

### 4.5 Schisandrin B in glioma

Glioma represents a group of heterogeneous brain tumors, accounting for 81% of malignant tumors within the central nervous system (Xu et al., 2020). Despite the development of numerous treatment approaches for glioma to date, the presence of the blood-brain barrier impedes the entry of most antitumor drugs into the brain, resulting in a scarcity of drugs currently used for the treatment of glioma (Ballabh et al., 2004; Oberoi et al., 2016). Research has shown that Sch B can inhibit the migration and invasion of glioma cells by suppressing the expression of p-Akt, p-mTOR, and MMP-9 in the PI3K/Akt-mTOR-MMP-9 signaling pathway (Jiang et al., 2015). Additionally, Sch B regulates the HOTAIR-miR-125a-mTOR pathway by reducing hox transcript antisense intergenic RNA (HOTAIR) expression and increasing miR-125a-5p expression to exert its function in suppressing the migration and invasion of glioma cell lines (Jiang et al., 2017). Moreover, Sch B induces cell cycle arrest of glioma cells in the G0/G1 phase, triggers apoptosis through the activation of caspase-3, caspase-9, poly (ADP-ribose) polymerase (PARP), and Bcl-2, and significantly inhibits the tumor growth of glioma cells *in vivo* (Li et al., 2015; Qi et al., 2016). Evidently, Sch B holds the potential for the treatment of glioma.

### 4.6 Schisandrin B in other tumors

In addition to the aforementioned cancer types, the antitumor effects and mechanisms of Sch B in other cancers have also been initially revealed in preclinical studies. Sch B has been demonstrated to inhibit the proliferation of human cholangiocarcinoma cells both *in vitro* and *in vivo* and to induce their apoptosis (Yang et al., 2016). Sch B can also trigger apoptosis of gallbladder cancer cells by modulating the expression of apoptosis-related proteins (up-regulating Bcl-2-associated X protein [Bax], cleaved caspase-9, cleaved caspase-3, cleaved PARP and down-regulating Bcl-2, NF-κB, cyclin D1 and cyclin-dependent kinase 4 [CDK-4]) (Xiang et al., 2014). These preclinical research results imply the potential application of Sch B in the treatment of biliary tract tumors. Over a decade ago, researchers discovered that Sch B was able to restrain the proliferation, migration and invasion of human lung adenocarcinoma A549 cells (Lv et al., 2015). Fu-Yi Cai et al. fabricated co-delivery liposomes featuring PFV-modified doxorubicin plus Sch B. The experimental results demonstrated that this targeted liposome had excellent targeting characteristics and was capable of suppressing the migration and invasion of non-small cell lung cancer cells, while simultaneously enhancing the cytotoxicity of doxorubicin and reducing its side effects, thereby providing a novel concept for the therapeutic approach of non-small cell lung cancer chemotherapy (Cai et al., 2021). RPV-modified paclitaxel and Sch B liposomes exhibit an anti-ovarian cancer effect by disrupting angiogenesis channels, inhibiting angiogenesis, and restraining the proliferation and migration of ovarian cancer cells (Zhang et al., 2021). Sch B remarkably induces apoptosis and autophagy in human head and neck squamous cell carcinoma cells. Notably, the inhibition of autophagy augments the apoptotic effect of Sch B (Li M. et al., 2024). In the early stage, osteosarcoma is often accompanied by lung metastasis. Sch B exerts its effect on the PI3K/Akt signaling pathway by modulating the circ_0009112/miR-708-5p axis, promoting the apoptosis of osteosarcoma cells and significantly suppressing the growth and lung metastasis of osteosarcoma in animal models (Wang et al., 2020; Wang et al., 2022). Researches have demonstrated that Sch B induces the apoptosis of prostate cancer cell lines by generating oxidative stress, suppressing androgen receptors, as well as the phosphorylation of PI3K/AKT and JAK2/STAT3 (Nasser et al., 2019). Furthermore, Sch B significantly reduces the cellular activity and malignant progression of melanoma cells by inhibiting the Wnt/β-catenin signaling pathway and exhibited favorable antitumor effects in both *in vitro* and *in vivo* experiments (Chen et al., 2023).

## 5 Prediction of potential targets of Schisandrin B in tumors

Most drugs exert their effects by means of the binding of drug molecules to biological macromolecules such as proteins in the body. At present, the action targets of many bioactive molecules are still unknown. Predicting the targets of drug molecules is of great significance for the development of new drugs and the exploration of new uses for old drugs (Gfeller et al., 2013). Researchers mainly employ two approaches, namely experimentation and computation, for target prediction. The experimental method has relatively high accuracy but is rather time-consuming and laborious. In the case of conducting a large number of screening tasks, the cost is extremely high. Conversely, computational prediction consumes relatively less time. However, the reliability of current algorithms is inferior to that of the experimental method. Generally, the results obtained through theoretical calculations need further verification by experiments (Zheng et al., 2004; Forouzesh et al., 2019). Existing research has shown that Sch B is a specific inhibitor of ataxia telangiectasia and rad3 - related (ATR) protein kinase (Nishida et al., 2009). The gomisin N (GN), which is the diastereoisomer of Sch B, inhibits DNA damage checkpoint signal transduction by interacting stereospecifically with ATR (Tatewaki et al., 2013). Pan et al. discovered that Sch B could reverse the drug resistance of multiple multidrug resistance cell lines overexpressing p-glycoprotein. Furthermore, the interaction between Sch B and p-glycoprotein could fully restore the intracellular drug accumulation, thereby proving that Sch B was a novel p-glycoprotein inhibitor (Qiangrong et al., 2005).

**FIGURE 1:**
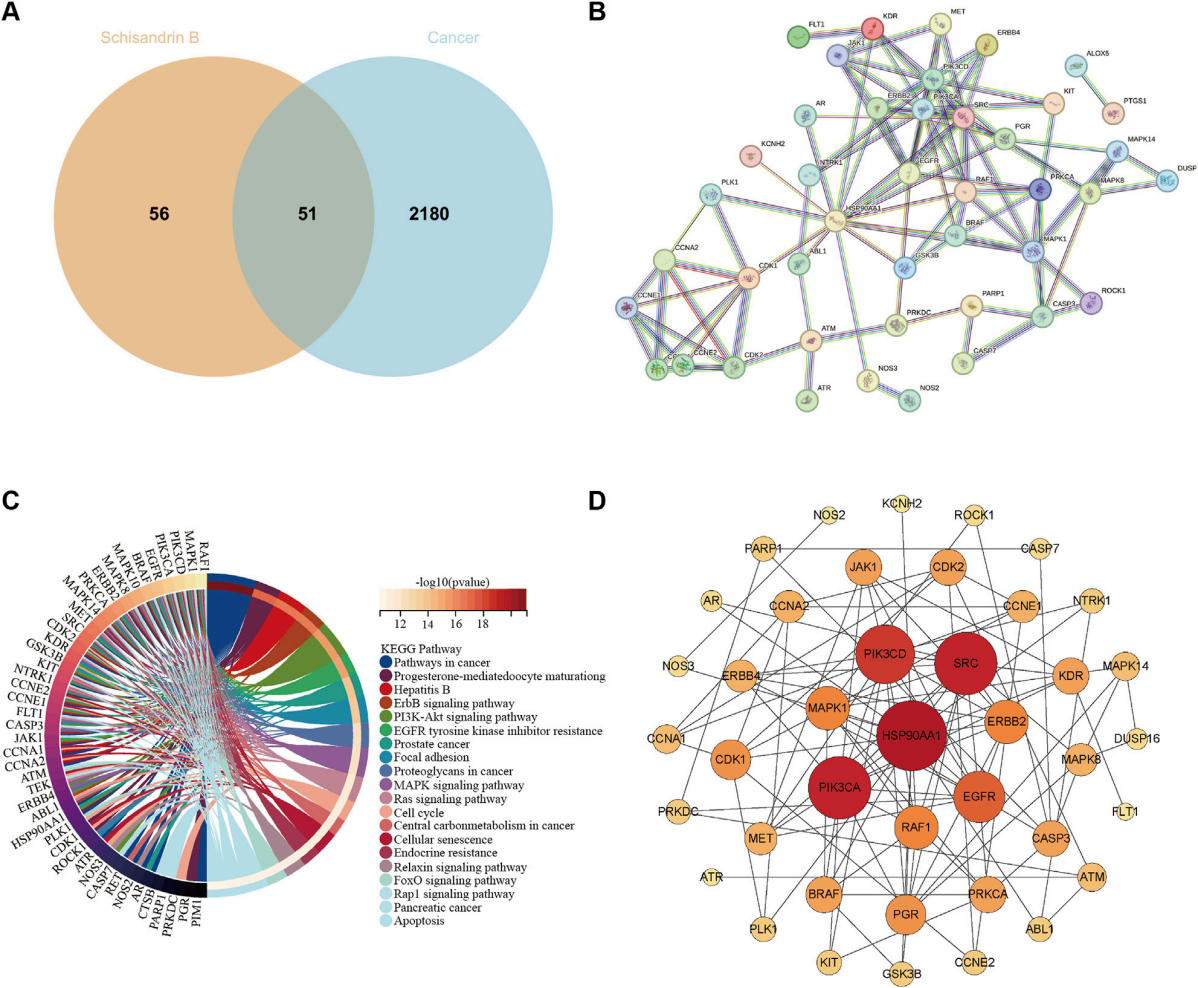
Predict the antitumor targets of Sch B. **(A)** The number of targets of Sch B acting on tumors. **(B)** The PPI network of 51 antitumor targets of Sch B. **(C)** The related signaling pathways of antitumor targets of Sch B. **(D)** The core targets of the antitumor effect of Sch B.

We employed a series of network pharmacology analysis methods to explore more potential targets of Sch B in tumor therapy. Initially, the targets of Sch B were retrieved from the SwissTargetPrediction database. We selected only those targets labeled “*homo sapiens*” for research (http://www.swisstargetprediction.ch/). Subsequently, on the Genecards database (https://www.genecards.org/), a search was conducted with “cancer and tumor” as the keyword and a relevance score greater than 10 was set to obtain tumor targets. By intersecting the outcomes of these two components, 51 targets of Sch B acting on tumors were ultimately obtained (Figure 1A). The 51 targets were used to establish a protein-protein interaction (PPI) network on the String database (http://string-db.org/) to observe the interactions among different targets and performing KEGG analysis. The disconnected nodes were concealed, and shared proteins with a minimum interaction score of 0.9 were selected. The results indicated that Sch B might exert its antitumor efficacy by influencing signal pathways related to cell cycle, apoptosis, cell migration and invasion, which is in accordance with the existing research findings (Nasser et al., 2020) (Figures 1B, C). To determine the core targets that play a crucial role in the entire target network, these targets were analyzed in depth on Cytoscape. According to the important parameter of degree, core targets such as heat shock protein 90 alpha family class A member 1 (HSP90AA1), phosphatidylinositol - 4,5 - bisphosphate 3 - kinase catalytic subunit alpha (PIK3CA), and proto - oncogene tyrosine - protein kinase Src (SRC) can be obtained. And ATR, which has been proven to be a target of Sch B, is also among them (Figure 1D). Although we have predicted the targets of Sch B in antitumor via network pharmacology, the limitation of this method is that the prediction results are all obtained based on the calculation of protein structural similarity. Whether it truly exerts its effect through these target remains to be confirmed by more experiments.

## 6 Conclusion

**FIGURE 2:**
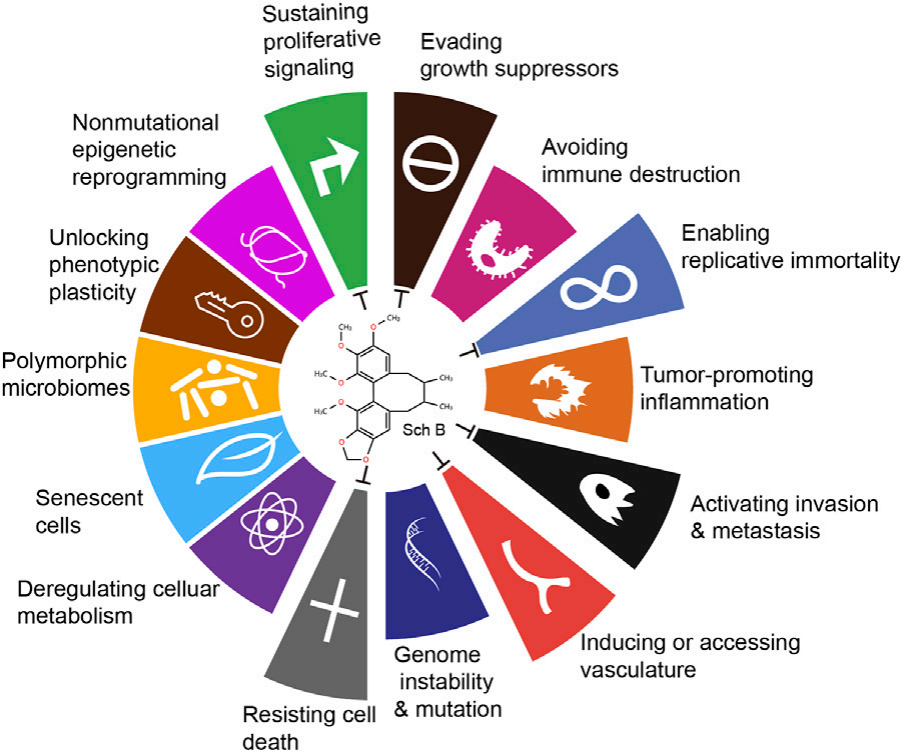
Antitumor effects of Sch B viewed from the fourteen characteristics of tumors.

Currently, surgery, radiotherapy and chemotherapy remain the main approaches for cancer treatment. Drawing on the current research findings on the antitumor mechanism of Sch B, it is evident that Sch B exerts its antitumor role through various means and pathways in preclinical studies. Douglas Hanahan and Robert A. Weinberg authoritatively released the fourteen major characteristics of tumors (Hanahan, 2022). Upon comparing the antitumor mechanism of Sch B, it is apparent that Sch B inhibits the progression of different tumors through six aspects, including: sustaining proliferative signaling, evading growth suppressors, enabling replicative immortality, activating invasion and metastasis, inducing or accessing vasculature, resisting cell death (Figure 2).

However, the current research on Sch B does reveal several potential limitations. Despite the identification of multiple antitumor targets of Sch B, as of now, there is a scarcity of relevant research reports on its clinical application. This deficiency significantly hampers the translation process from basic research to clinical implementation. Traditional treatment modalities, such as surgical intervention, often enable radical resection of early-stage localized tumors. During extensive long-term clinical practice, radiotherapy and chemotherapy have accumulated substantial experience and demonstrated well-defined curative efficacy in a diverse range of tumors. In sharp contrast, the clinical evidence related to Sch B remains relatively limited. Current research results are insufficient to rule out the potential for long-term or high-dose administration of Sch B to induce toxicity in specific organs or systems. This may include adverse effects on the functions of crucial organs like the liver and kidneys, as well as potential impacts on the immune system. Additionally, the current research on the interactions between Sch B and existing clinical medications is still inadequate. This deficiency has the potential to undermine the safety and efficacy of combination therapies.

Undoubtedly, as researchers embark on a more systematic exploration of Sch B’s toxicity characteristics with the aim of ensuring its safe clinical application, delving deeper into its formulation technology and developing a more efficient, stable, and targeted drug delivery system to enhance its bioavailability and therapeutic efficacy, they will also need to assess the efficacy and safety of Sch B across diverse tumor types and stages. Simultaneously, clarifying its optimal combination regimens and the appropriate patient populations is of crucial importance. On the other hand, whether Sch B can enhance the efficacy of radiotherapy and act as a radiation sensitizer is also a research direction worthy of in-depth exploration by researchers. Through these efforts, the application of Sch B in the antitumor field is expected to expand in both scope and depth. Consequently, Sch B is anticipated to emerge as a potential component of novel antitumor medications, thus offering new hope to cancer patients.
